# Overcoming NK cell resistance in triple-negative breast cancer via adcc with a humanized anti-CD147 antibody

**DOI:** 10.1007/s00262-025-04203-z

**Published:** 2025-10-22

**Authors:** Thanathat Pamonsupornwichit, Kanyarat Thongheang, Nuchjira Takheaw, Kanokporn Sornsuwan, Zaw Ye Htet, On-anong Juntit, Phatcharida Jantaree, Chatchai Tayapiwatana

**Affiliations:** 1 Center of Biomolecular Therapy and Diagnostic, Faculty of Associated Medical Sciences, Chiang Mai University, Chiang Mai, 50200 Thailand; 2 Division of Clinical Immunology, Department of Medical Technology, Faculty of Associated Medical Sciences, Chiang Mai University, Chiang Mai, 50200 Thailand; 3 Biomedical Technology Research Center, National Center for Genetic Engineering and Biotechnology, National Science and Technology Development Agency, Faculty of Associated Medical Sciences, Chiang Mai University, Chiang Mai, 50200 Thailand; 4 Office of Research Administration, Chiang Mai University, Chiang Mai, 50200 Thailand; 5 Center of Multidisciplinary Technology for Advanced Medicine (CMUTEAM), Faculty of Medicine, Chiang Mai University, Chiang Mai, 50200 Thailand

**Keywords:** Breast cancer, TNBC, CD147, Humanized antibody, ADCC, NK cell, Immunotherapy

## Abstract

Triple-negative breast cancer (TNBC) is an aggressive and clinically challenging subtype defined by the absence of estrogen receptor, progesterone receptor, and HER2 amplification, resulting in poor prognosis and limited therapeutic options. Targeting alternative molecular pathways is urgently needed to overcome resistance and improve patient outcomes. CD147 has emerged as surface marker associated with tumor progression and immune evasion. In this study, CD147 and MHC class I—a key inhibitory ligand for natural killer cells—were analyzed in breast cancer cell lines (MCF7, MDA-MB-453, MDA-MB-231, and HCC38) using flow cytometry. The therapeutic efficacy of a humanized anti-CD147 monoclonal antibody (HuM6-1B9) was evaluated for its capacity to potentiate antibody-dependent cellular cytotoxicity (ADCC). HuM6-1B9 demonstrated the strong binding to MDA-MB-231 (*K*_D_ = 4.982 nM) and HCC38 (*K*_D_ = 4.523 nM), which are representative TNBC cell lines. In 3D spheroid models, HuM6-1B9 significantly enhanced PBMC-mediated ADCC, leading to a marked reduction in TNBC spheroid viability. Co-culture of CFSE-labeled MDA-MB-231 and HCC38 cells with primary NK cells confirmed robust ADCC, achieving 50% and 70% cytotoxicity, respectively, despite high MHC class I expression. Live-cell imaging demonstrated caspase-3/7 activation consistent with apoptosis in NK-targeted tumor cells, while CD107a degranulation and IFN-γ secretion confirmed the functional contribution of HuM6-1B9 to ADCC enhancement. Importantly, HuM6-1B9 did not promote migration or invasion in MDA-MB-231 cells, supporting its safety profile regarding metastasis. Collectively, these findings establish HuM6-1B9 as a promising immunotherapeutic candidate that overcomes immune resistance and selectively eliminates TNBC cells through ADCC without enhancing metastatic potential. By integrating mechanistic assays of NK cytotoxicity, apoptosis, and 3D tumor spheroids, this study provides clinically relevant insights underscoring the translational potential of HuM6-1B9 in TNBC immunotherapy.

**Supplementary Information:**

The online version contains supplementary material available at 10.1007/s00262-025-04203-z.

## Introduction

Breast cancer constitutes a major global health concern, representing the most frequently diagnosed malignancy among women [1]. Despite conventional therapeutic modalities encompassing surgery, radiation, and drug therapy, the persistent challenges of cancer recurrence and metastasis contribute to unfavorable patient prognoses [2]. Triple-negative breast cancer (TNBC), a particularly aggressive subtype, presents a significant clinical obstacle. Characterized by the absence of estrogen receptor (ER), progesterone receptor (PR), and human epidermal growth factor receptor 2 (HER2), making it unresponsive to standard hormone therapies and HER2-targeted treatments like trastuzumab [3, 4]. Notwithstanding the identification of potential therapeutic targets, TNBC remains a considerable clinical challenge [5]. Beyond conventional therapeutic targets, other molecules such as CD146 [6, 7] and CD73 have been explored in TNBC [8, 9]. Elevated CD146 expression has been linked to tumor progression, although interpatient variability limits its therapeutic applicability [10]. CD73 contributes to immune evasion through adenosine generation, and antibody–drug conjugates targeting CD73 have shown preclinical efficacy [11]. However, their direct role in tumor cell survival remains less pronounced.

Another emerging therapeutic target in breast cancer is CD147, a transmembrane glycoprotein that is markedly overexpressed in breast cancer tissues and is associated with poor clinical outcomes [12, 13]. The oncogenic potential of CD147 is largely attributed to its role in promoting breast cancer cell migration and invasion, primarily through the activation of the MAPK/ERK signaling pathway and the induction of epithelial–mesenchymal transition (EMT) [14]. In addition, CD147 has been shown to interact with CD276 in cancer stem cells, a mechanism that contributes to chemoresistance and worsened prognosis [15]. Beyond its functions in tumor progression, CD147 also modulates the expression of vacuolar H⁺-ATPase and drug efflux transporters, thereby enhancing resistance to chemotherapeutic agents in breast cancer [16, 17]. Owing to its multifaceted involvement in tumor progression and therapeutic resistance, CD147 holds promise as both a prognostic biomarker and a novel therapeutic target in breast cancer management.

Natural killer (NK) cells, a critical component of the innate immune system, play a fundamental role in antitumor immunity by mediating the lysis of MHC class I-deficient cancer cells. This is significant as MHC class I typically functions as an inhibitory ligand for NK cells. However, certain cancer cells can upregulate MHC class I expression in response to NK cell activity, thereby facilitating tumor evasion from NK cell-mediated cytotoxicity [18, 19]. Antibody-dependent cell-mediated cytotoxicity (ADCC) represents a key mechanism to counteract this evasion. ADCC is an immune mechanism that enhances antitumor activity by activating immune cells, such as NK cells, to specifically target and destroy antibody-coated cancer cells [20].

Recently, a humanized antibody targeting CD147 (HuM6-1B9) was developed to minimize immunogenicity for therapeutic use [21]. This antibody has demonstrated efficacy in eliminating T-cell acute lymphoblastic leukemia (T-ALL) cells by enhancing antibody-dependent cellular phagocytosis (ADCP) [21]. However, in breast cancer, the overexpression of CD47—a "don't eat me" signal—may inhibit macrophage-mediated phagocytosis, potentially limiting the effectiveness of ADCP-based strategies [22, 23]. Given this challenge, it is important to evaluate whether HuM6-1B9 can activate alternative immune mechanisms, such as ADCC, to mediate antitumor effects. Previously, our data demonstrated that HuM6-1B9 potentially promotes PBMC-mediated cytotoxicity against the MDA-MB-231 cell line [24], which represents the TNBC subtype. However, the ADCC activity of HuM6-1B9 in a three-dimensional (3D) Matrigel-based culture model of TNBC cell line that mimics the in vivo extracellular matrix of a tumor has not yet been investigated.

In this study, we assessed the expression of CD147, CD146, CD73, and MHC class I across a panel of breast cancer cell lines representing triple-negative (MDA-MB-231, and HCC38), hormone receptor-positive (MCF7), and HER2-positive (MDA-MB-453) subtypes. We then investigated the therapeutic potential of HuM6-1B9 in enhancing PBMC-mediated ADCC against TNBC spheroids and NK cell-mediated ADCC against breast cancer subtypes. Furthermore, we examined the functional effects of CD147 targeting by HuM6-1B9 on MDA-MB-231 cell migration and invasion. Collectively, our findings aim to support the development of novel molecular targets and immunotherapeutic strategies for the treatment of TNBC.

## Materials and methods

### Cell lines

Breast cancer cell lines, including MDA-MB-231 (clone HTB-26), MCF7 (clone HTB-22), MDA-MB-453 (HTB-131), and HCC38 (clone CRL-2314), were purchased from American Type Culture Collection (ATCC). Human embryonic kidney 293T (HEK293T) cell line was kindly provided by Prof. Dr. Watchara Kasinrerk, Faculty of Associated Medical Sciences, Chiang Mai University, Chiang Mai, Thailand. The MDA-MB-231, MCF7, MDA-MB-453, and HEK293T cell lines were maintained in Dulbecco’s Modified Eagle Medium (DMEM) (Gibco, Carlsbad, CA, USA) supplemented with 10% heat-inactivated fetal bovine serum (FBS) (Gibco, Carlsbad, CA, USA), 100 U/mL of penicillin, and 100 µg/mL of streptomycin (Gibco, Carlsbad, CA, USA). HCC38 cell line was cultured in Roswell Park Memorial Institute (RPMI) 1640 (ATCC) supplemented with 10% FBS, 100 U/mL of penicillin, and 100 µg/mL of streptomycin.

### Preparation of HuM6-1B9

The HuM6-1B9 was produced in our laboratory as previously described [24]. Briefly, the pVITRO1-HuM6-1B9-IgG1/κ plasmid [21] was transfected in HEK293T cells. Transfected cells were selected with 400 µg/mL hygromycin B (InvivoGen, San Diego, CA, USA) and subsequently adapted to HyClone CDM4HEK293 serum-free medium (Cytiva, Uppsala, Sweden). For large-scale production, the adapted cells were inoculated in a bioreactor flask and maintained in HyClone CDM4HEK293 serum-free medium supplemented with 400 μg/mL hygromycin B. The culture supernatant containing HuM6-1B9 was then collected and subjected to purification using HiTrap™ Protein G affinity chromatography (Cytiva, Uppsala, Sweden).

### Binding affinity and cell surface expression using flow cytometric analysis

To assess the binding affinity of purified HuM6-1B9, human breast cancer cell lines, MDA-MB-231, MCF7, MDA-MB-453, and HCC38 were harvested and resuspended in fluorescence-activated cell sorting (FACS) buffer containing 20% FBS. The cells were incubated on ice for 30 min to block nonspecific binding. Subsequently, cells were incubated with various concentrations of HuM6-1B9 on ice for 30 min. After three washes with FACS buffer, cells were incubated for an additional 30 min on ice with PE-conjugated goat anti-human IgM/IgG/IgA F(ab′)₂ secondary antibody (1:250 dilution; Merck Millipore, Darmstadt, Germany). Following a final series of washes, fluorescence was analyzed using a BD Accuri C6 Plus flow cytometer (BD Biosciences, Franklin Lakes, NJ, USA). Data acquisition and analysis were performed using FlowJo software (BD Biosciences).

To compare the surface expression levels of CD147, CD146, CD73, and MHC class I across various breast cancer cell lines, cells were first incubated in FACS buffer supplemented with 10% heat-inactivated human AB serum for 30 min on ice. Following this, cells were stained with the following primary antibodies: mouse anti-CD147 mAb, clone M6-1B9 (produced in-house); mouse anti-human CD146 mAb, clone P1H12 (BioLegend, San Diego, CA, USA); mouse anti-human CD73 mAb, clone AD2 (BioLegend, San Diego, CA, USA); and mouse anti-human MHC class I mAb, clone MEM-147 (ImmunoTools, Friesoythe, Germany). After primary staining, cells were washed and incubated with FITC-conjugated F(ab′)₂ goat anti-mouse IgG + IgM (H + L) secondary antibody (ImmunoTools, Friesoythe, Germany) for 30 min on ice. A conjugated control was included by staining cells with the secondary antibody alone. Stained cells were analyzed using a BD Accuri C6 Plus flow cytometer, and data were processed using FlowJo software.

### Assessment of ADCC activity on TNBC spheroids

MDA-MB-231 cells (5 × 10^3^ cells/well) were suspended in 2% Matrigel (Corning, NY, USA) and seeded into a 96-well round-bottom, ultra-low attachment plate (Corning, NY, USA). The plate was incubated at 37 °C in a 5% CO₂ incubator for 72 h to allow spheroid formation (single spheroids in each well). Peripheral blood mononuclear cells (PBMCs) were isolated from heparinized peripheral blood sample using Lymphoprep™ (STEMCELL Technologies, Vancouver, Canada) as a density gradient medium. To assess ADCC activity, PBMCs (5 × 10^4^ cells) were co-cultured with TNBC spheroids in the presence or absence of HuM6-1B9 (10 μg/mL) and incubated under the same conditions for an additional 72 h. Cisplatin (20 μM; MedChemExpress, NJ, USA) was used as a positive control to confirm spheroid responsiveness to cytotoxic agents. Following incubation, the cell viability was determined using PrestoBlue™ Cell Viability Reagent (Thermo Fisher Scientific, Waltham, MA, USA), in accordance with the manufacturer’s instructions.

### Live/Dead assay on TNBC spheroids

TNBC spheroids were generated and co-cultured with PBMCs in the presence or absence of HuM6-1B9 (10 μg/mL), as previously described. After 72 h of incubation, spheroids were stained using the LIVE/DEAD™ Viability/Cytotoxicity Kit (Thermo Fisher Scientific, Waltham, MA, USA), following the manufacturer’s protocol. Stained spheroids were analyzed using the Operetta CLS™ high-content analysis system (Revvity, Waltham, MA, USA), and the extent of cell death was quantified as the ratio of dead to live stained cells. Additionally, spheroid imaging was performed using a confocal microscope (Olympus FV3000, Japan) at 100× magnification to visualize treatment effects.

### Assessment of PBMC Infiltration into TNBC Spheroids

MDA-MB-231 cells were pre-labeled with Hoechst 33342 (Thermo Fisher Scientific, Waltham, MA, USA) prior to spheroid formation. PBMCs were stained with CMFDA dye (Thermo Fisher Scientific, Waltham, MA, USA), and introduced to the TNBC spheroids after 72 h of spheroid formation, in the presence or absence of HuM6-1B9 (10 μg/mL). Spheroids cultured without PBMCs served as negative controls. PBMC infiltration into the spheroids was evaluated using the Operetta CLS™ High-Content Analysis System.

### ADCC assay by primary NK cells

Human breast cancer cell lines were detached using Accutase solution (Gibco, Waltham, MA, USA) and subsequently labeled with 2 µM carboxyfluorescein diacetate succinimidyl ester (CFSE) (Thermo Fisher Scientific, Waltham, MA, USA) to serve as target cells. Primary NK cells were isolated from heparinized peripheral blood samples collected from healthy donors using the EasySep™ Human NK Cell Isolation Kit (STEMCELL Technologies, Vancouver, Canada) and were used as effector cells. The purity of the isolated NK cells was assessed by flow cytometry based on CD3⁻CD56⁺ expression. For the ADCC assay, CFSE-labeled target cells (2 × 10^4^ cells/well) were pre-incubated for 10 min at room temperature with either HuM6-1B9, purified human immunoglobulin G (hIgG) as antibody control, or medium alone (no antibody control). Following antibody incubation, NK cells were added to the target cells at an effector-to-target (E:T) ratio of 3:1. Wells without effector cells and antibody were included as controls for determining spontaneous target cell death. Co-cultures were established in 96-well U-bottom plates (Thermo Fisher Scientific) and incubated at 37 °C in a 5% CO₂ incubator for 4 h (MDA-MB-231, MDA-MB-453, and HCC38) or 2 h (MCF7). After incubation, cells were harvested and stained with 5 µg/mL propidium iodide (PI) (Sigma-Aldrich, St. Louis, MO, USA) for 10 min at room temperature in the dark. The percentage of dead target cells (CFSE⁺PI⁺) was quantified using a BD Accuri C6 Plus flow cytometer, and data were analyzed using FlowJo software.

### Assay for CD107a (LAMP-1) degranulation

For determination of NK cell degranulation, the isolated NK cells were incubated with MDA-MB-231 target cell line (2 × 10^4^ cells/well) at E:T ratio of 3:1. The co-cultured cells were incubated with hIgG or HuM6-1B9 at 10 μg/mL or without antibody treatment in RPMI-1640 containing 10% FBS, 0.1 μM monensin, and PE-anti-LAMP-1 mAb (BD Biosciences, Franklin Lakes, NJ, USA) or PE-mouse IgG1 mAb (ImmunoTools, Friesoythe, Germany) as isotype control. The co-cultured cells were incubated at 37 °C in a 5% CO_2_ incubator for 4 h. Following incubation, the cells were harvested and stained with PE/Cy5-anti-CD56 mAb, clone HCD56 (BioLegend, San Diego, CA, USA). The percentage of NK cell degranulation (CD107a-PE^+^ CD56-PE/Cy5^+^) was acquired using BD Accuri C6 Plus flow cytometer and analyzed using FlowJo software.

### Interferon-gamma (IFN-γ) release assay

MDA-MB-231 target cells (2 × 10^4^ cells/well) were co-cultured with isolated NK cells in 96-well U-bottom plates at E:T ratio of 3:1, in the presence of antibody (hIgG or HuM6-1B9 at 10 μg/mL) or absence of antibody. The co-cultured cells were incubated at 37 °C in a 5% CO_2_ incubator for 4 h. After incubation, the culture supernatants were collected to measure the IFN-γ levels using a Human IFN-γ ELISA Kit (FineTest®, Wuhan, China).

### Fcγ receptor III (FcγRIII) blocking assay

To assess whether HuM6-1B9-mediated NK cell cytotoxicity was dependent on FcγRIIIa (CD16a), the blocking CD16 on NK cells was performed. Primary NK cells were incubated with anti-CD16 mAb clone 3G8 (Bio-Rad, CA, USA) for 30 min at room temperature. Following the blocking step, the NK cells were co-cultured with CFSE-labeled MDA-MB-231 target cells (E:T ratio of 3:1) in the medium control, hIgG or HuM6-1B9 at 10 μg/mL and incubated at 37 °C in a 5% CO_2_ incubator for 4 h. After incubation, the co-cultured cells were harvested and stained with PI at 5 µg/mL. The percentage of dead target cells (CFSE⁺PI⁺) was quantified using a BD Accuri C6 Plus flow cytometer, and data were analyzed using FlowJo software.

### Assessment of an ADCC by the combination of HuM6-1B9 and immune checkpoint inhibitor

To evaluate whether the combination of HuM6-1B9 and immune checkpoint blockade enhances NK cell-mediated cytotoxicity against TNBC cell line, pembrolizumab (anti-PD-1 mAb; KEYTRUDA®) was employed. Primary NK cells were pre-treated with pembrolizumab at a concentration of 10 µg/mL prior to co-culture with CFSE-labeled MDA-MB-231 target cells at an E:T ratio of 3:1. Co-cultures were conducted in the presence of either hIgG, HuM6-1B9 (5 µg/mL), or without antibody treatment. The cells were incubated at 37 °C in a 5% CO₂ incubator for 4 h. Following incubation, cells were stained with PI at 5 µg/mL to assess cytotoxicity. The percentage of dead target cells (CFSE⁺PI⁺) was quantified using a BD Accuri C6 Plus flow cytometer, and data were analyzed using FlowJo software.

### MHC-Class I chain-related proteins A and B (MIC-A/B) expression assay

To determine the modulation of HuM6-1B9 on MIC-A/B expression on TNBC cell line, MDA-MB-231 cells (5 × 10^4^ cells) were seeded to 96-well flat-bottom plate in the presence of hIgG or HuM6-1B9 at 10 µg/mL and incubated at 37 °C in a 5% CO₂ incubator for 24 h. After incubation, the cells were detached with Accutase solution and subsequently stained with APC anti-human MIC-A/B mAb, clone 6D4 (BioLegend, San Diego, CA, USA) for 30 min on ice. Stained cells were analyzed using a BD Accuri C6 Plus flow cytometer, and data were processed using FlowJo software.

### Live-cell imaging analysis for apoptosis determination

MDA-MB-231 cells (1 × 10^4^ cells/well) were seeded in 96-well, black, flat-bottom plate (PerkinElmer, Waltham, MA, USA) in 10% FBS DMEM and incubated overnight at 37 °C in a 5% CO₂ incubator. Following incubation, the breast cancer cells were stained with PhenoVue 505 live-cell caspase-3/7 activity (Revvity, Waltham, MA, USA) at a final concentration of 5 μM for 30 min at 37 °C in a 5% CO₂ incubator. Primary NK cells were then co-cultured with stained breast cancer cells in the presence or absence of HuM6-1B9 or hIgG. To assess breast cancer cell apoptosis, live-cell imaging was performed using an Operetta CLS™ high-content analysis system at 100× magnification.

### Transwell migration assay

Cell migration assay was performed in 24-well plates equipped with 8 µm pore sized chamber inserts (Wuxi NEST Biotechnology, Wuxi, Jiangsu, China). MDA-MB-231 cells (4 × 10^4^ cells/well) were resuspended in 100 µL of DMEM containing HuM6-1B9 or hIgG as a control, at concentrations of 1 and 10 μg/mL. These cell suspensions were then seeded into the upper chamber. The lower chambers were filled with 600 µL of DMEM supplemented with 10% FBS (C-Medium). Plates were incubated for 24 h at 37 °C in 5% CO_2_ incubator. Following incubation, cells remaining on the upper surface of the membrane were gently removed using a moist cotton swab. Cells that migrated to the lower surface of the membranes were fixed with methanol and subsequently stained with Wright-Giemsa. Quantification of migratory cells was counted under ZEISS Axioscope 5 microscope (Carl Zeiss Microscopy, LLC., NY, USA) at 100× magnification.

### Cell invasion assay

The invasion assay was conducted using BD Matrigel™ Invasion chamber in 24-well plates with 8 µm pore size inserts (BD Biosciences, MA, USA). MDA-MB-231 cells were cultured in DMEM for 24 h prior to the initiation of the assay. Following this pre-incubation, MDA-MB-231 cells (5 × 10^4^ cells/well) were resuspended in 100 µL of DMEM containing HuM6-1B9 or hIgG as a control. These cell suspensions were seeded into the upper chamber of the inserts. The lower chambers were filled with 600 µL of C-Medium. The plates with chambers were incubated for 22 h at 37 °C in 5% CO_2_ incubator. Subsequently, cells remaining on the upper surface were carefully removed using a moist cotton swab. The invasive cells were fixed with methanol and stained with Wright-Giemsa. The number of invasive cells was counted under ZEISS Axioscope 5 microscope at 100× magnification.

### Statistical analysis

All experiments were performed in triplicate or quadruplicate. Data were analyzed using GraphPad Prism software, version 10.1.0 (GraphPad Software, San Diego, CA, USA). One-way analysis of variance (ANOVA) followed by Tukey’s multiple comparison test or Dunnett’s post hoc test was used for group comparisons. Two-way ANOVA followed by Fisher’s test was utilized for two-independent variables between group comparisons. Statistical significance was defined as followed: ns, *P* > 0.05; *, *P* ≤ 0.05; **, *P* ≤ 0.01; ***, *P* ≤ 0.001; ****, *P* ≤ 0.0001.

## Results

### Differential surface expression of CD147, CD146, CD73, and MHC class I in breast cancer cell lines

**Fig. 1:**
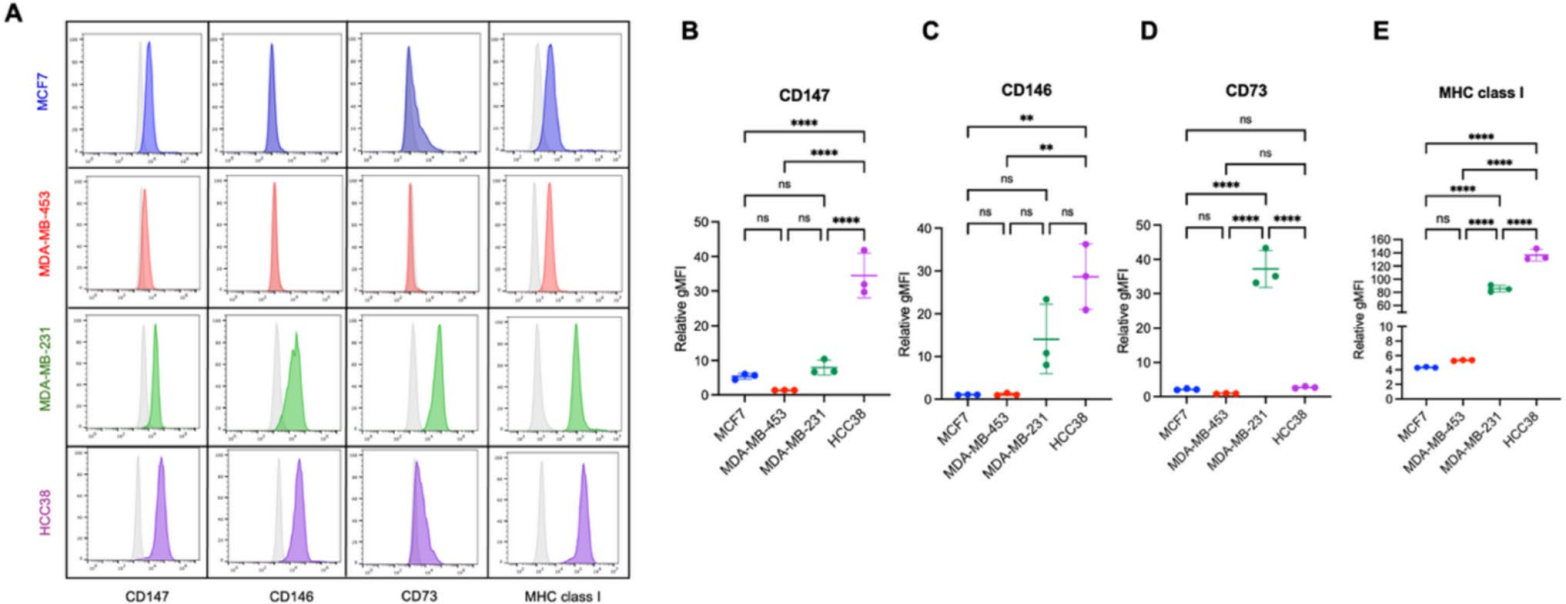
Expression profiles of CD147, CD146, CD73, and MHC class I in breast cancer cell lines. Breast cancer cells were stained with mouse mAbs specific for CD147, CD146, CD73, and MHC class I, followed by FITC-conjugated F(ab')_2_ goat anti-mouse IgG + IgM (H + L). (**A**) Representative surface expression profiles for each cell line (filled histograms: blue for MCF7, red for MDA-MB-453, green for MDA-MB-231, and purple for HCC38) are shown in comparison with the conjugated control (gray histogram). (**B–E**) Relative gMFI (mean ± SD) of CD147, CD146, CD73, and MHC class I, respectively, across the four cell lines. The experiment was performed in triplicate. Statistical analysis was performed using one-way ANOVA followed by Tukey's multiple comparison test. Significance levels are indicated as follows: ns, *P* > 0.05; **P ≤ 0.01; ****P ≤ 0.0001

Breast cancer cell lines, including MCF7, MDA-MB-453, MDA-MB-231, and HCC38, were harvested and stained with mouse mAbs against CD147, CD146, CD73, and MHC class I. A FITC-conjugated F(ab′)₂ goat antimouse IgG + IgM (H + L) secondary antibody was used for detection. Flow cytometric analysis revealed that CD147, CD73, and MHC class I—but not CD146—were expressed on the surface of MCF7 cells. In contrast, MDA-MB-453 cells expressed only CD147 and MHC class I. Notably, all four markers were detected on MDA-MB-231, and HCC38 TNBC cells. The distinct expression profiles of each cell line are shown in Fig. 1A. To compare expression levels across cell lines, the relative geometric mean fluorescence intensity (gMFI) was calculated by normalizing the gMFI of antibody-stained cells to that of cells stained with the secondary antibody alone (conjugated control). CD147 was consistently expressed across all tested cell lines, with comparable levels observed in MCF7, MDA-MB-453, and MDA-MB-231, but showed the highest level in HCC38 cells (Fig. 1B). CD146 expression was uniquely detected in TNBC cell lines (MDA-MB-231, and HCC38) (Fig. 1C). CD73 showed the highest expression in MDA-MB-231 cells, whereas its expression was minimal in MCF7, and HCC38 cells (Fig. 1D). Interestingly, MHC class I—a critical inhibitory ligand for NK cells—was most abundantly expressed in TNBC cell lines with the highest expression in HCC38 cells compared to the other cell lines (Fig. 1E).

### Binding affinity analysis of HuM6-1B9 to breast cancer cell lines

**Fig. 2:**
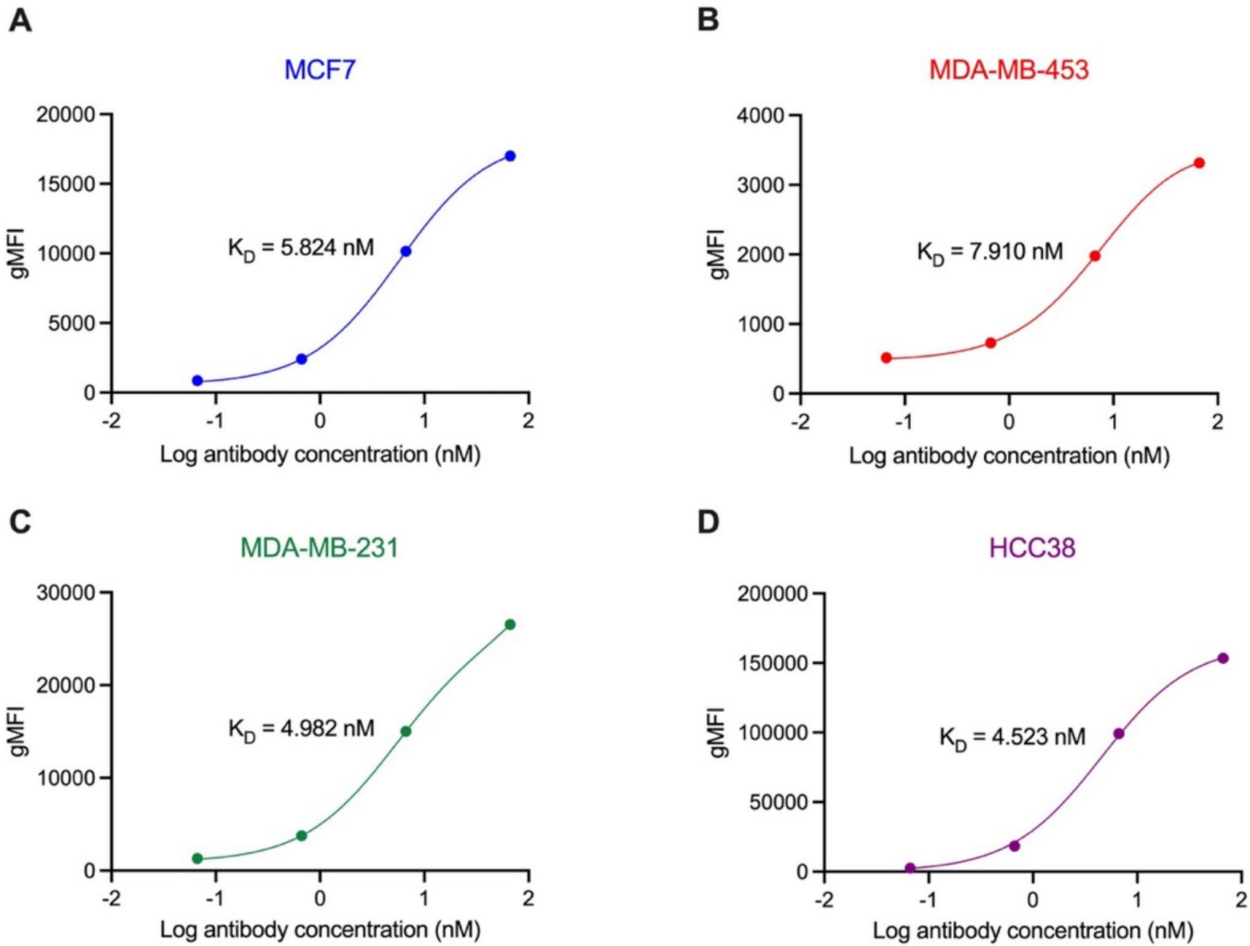
Affinity determination of HuM6-1B9 on breast cancer cell lines. Breast cancer cells were stained with various concentrations of HuM6-1B9 and analyzed by flow cytometry. The assay was conducted in triplicate, and the binding curves were generated using nonlinear regression. The K_D_ values were determined for (**A**) MCF7, (**B**) MDA-MB-453, (**C**) MDA-MB-231, and (**D**) HCC38 cell lines

The binding affinity of HuM6-1B9 for breast cancer cell lines was determined using a flow cytometry-based titration assay. gMFI was measured at various antibody concentrations, and binding curves for MCF7 (Fig. 2A), MDA-MB-453 (Fig. 2B), MDA-MB-231 (Fig. 2C), and HCC38 (Fig. 2D) cells were generated by nonlinear regression to calculate the dissociation constant (K_D_). This analysis revealed K_D_ values of 5.842 nM for MCF7 cells, 7.910 nM for MDA-MB-453 cells, 4.982 nM for MDA-MB-231 cells, and 4.523 nM for HCC38, indicating a high binding affinity of HuM6-1B9 on these breast cancer cell lines.

### HuM6-1B9 promotes PBMC-mediated cytotoxicity against TNBC spheroids

**Fig. 3:**
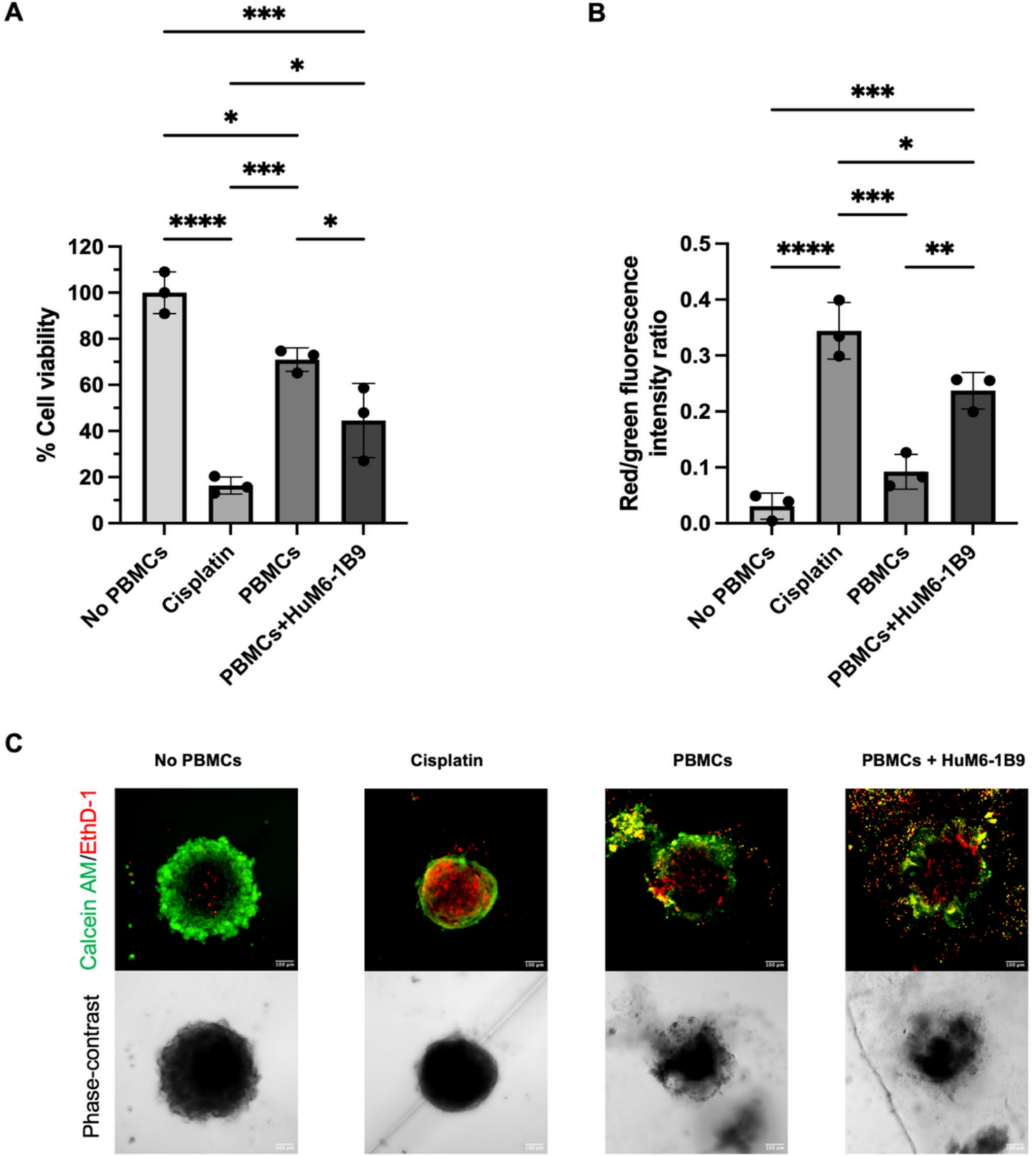
HuM6-1B9 enhanced the PBMC-induced cell death. (**A**) Cell viability (%) (mean ± SD) of TNBC spheroids was assessed after 72 h of treatment using the resazurin assay. Cell viability was calculated as the percentage ratio of the optical density of resorufin formed in treated cells compared to untreated control cells (no PBMCs). (**B**) Cell death of TNBC spheroids was assessed after 72 h of treatment. The spheroids were stained using the LIVE/DEAD™ Viability/Cytotoxicity Kit and analyzed with the Operetta CLS™ high-content analysis system. Cell death was expressed as a ratio of red-to-green fluorescence intensity (mean ± SD). (**C**) Representative confocal images of TNBC spheroids after 72 h of treatment (Scale bar = 100 μm). Assay was carried out in triplicate, and statistical analysis was assessed by one-way ANOVA followed by Tukey's multiple comparison test: *P ≤ 0.05; **P ≤ 0.01; ***P ≤ 0.001; ****P ≤ 0.0001

TNBC spheroids were established by embedding MDA-MB-231 cells in Matrigel. PBMCs were subsequently introduced into the spheroids in the presence or absence of HuM6-1B9. After 72 h of co-culture, spheroid viability was assessed using the resazurin assay. The results demonstrated that HuM6-1B9 markedly enhanced PBMC-mediated cytotoxicity, as indicated by a significant reduction in cell viability (%) (Fig. 3A). To further assess treatment effects, live/dead-cell staining was performed, with green and red fluorescence indicating viable and nonviable cells, respectively. As shown in Fig. 3B and C, HuM6-1B9 treatment substantially increased the red-to-green fluorescence intensity ratio, confirming its efficacy in promoting spheroid cell death in the presence of PBMCs. The assessment of PBMC infiltration into TNBC spheroids is shown in Supplementary Fig. S1.

### HuM6-1B9 enhances NK cell-mediated cytotoxicity against breast cancer cells

Breast cancer cells were labeled with CFSE and co-cultured with primary NK cells at E:T ratio of 3:1 in the presence of HuM6-1B9, hIgG, or medium alone. NK cell-mediated cytotoxicity was assessed by flow cytometry based on the percentage of CFSE^+^PI^+^. Gating strategy is shown in Supplementary Fig. S2.

**Fig. 4:**
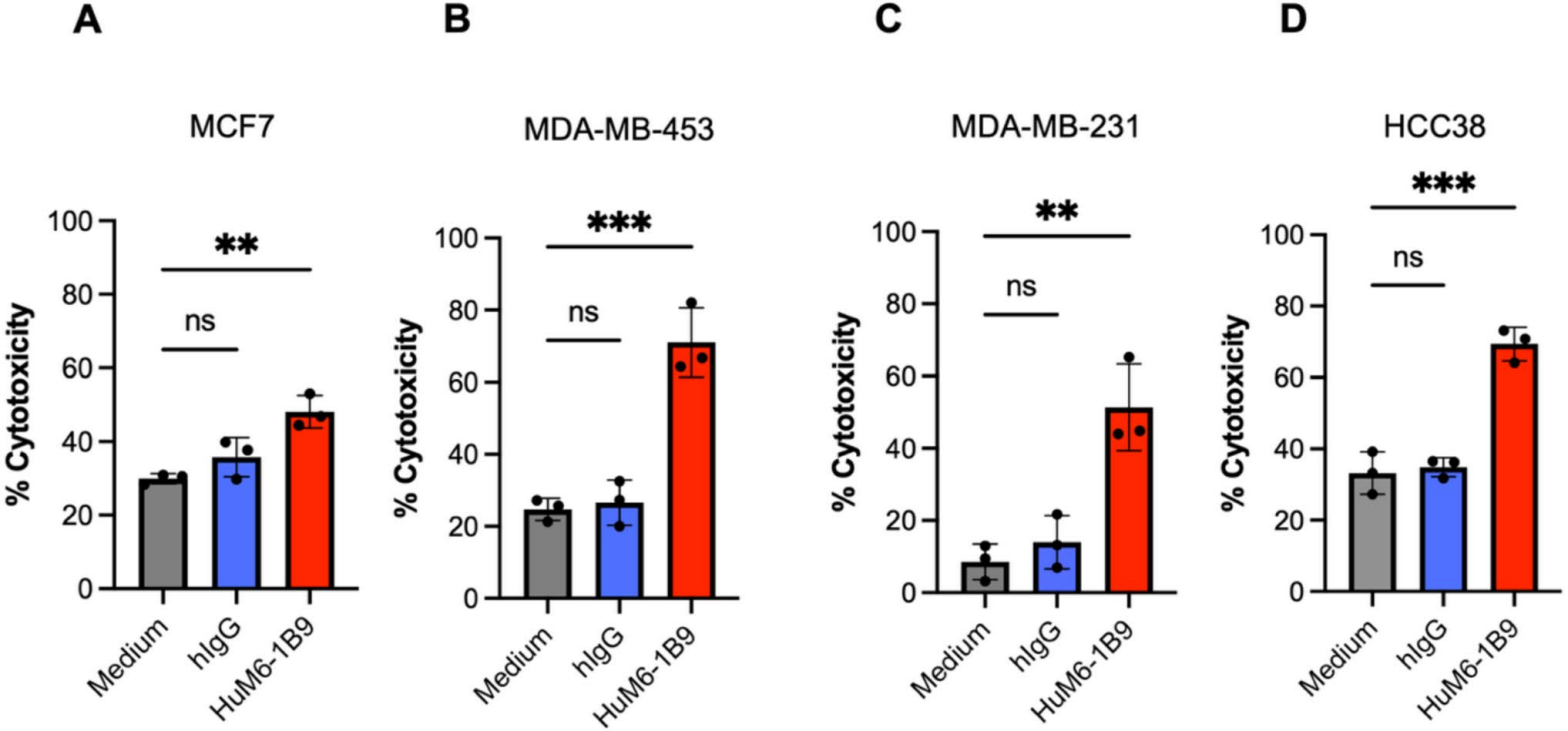
HuM6-1B9 enhances NK cell-mediated cytotoxicity against breast cancer cells via ADCC. CFSE-labeled (**A**) MCF7, (**B**) MDA-MB-453, (**C**) MDA-MB-231, and (**D**) HCC38 cells were co-cultured with primary NK cells at a 3:1 E:T ratio in the presence of HuM6-1B9, hIgG, or medium alone. Target cell death was quantified by flow cytometry based on CFSE and PI staining. Cytotoxicity (%) was calculated as [(% dead target cells – % spontaneous death) / (100 – % spontaneous death)] × 100. Data represent mean ± SD from three independent experiments. Statistical significance was determined by one-way ANOVA with Dunnett’s post hoc test: ns, *P* > 0.05; **P ≤ 0.01; ***P ≤ 0.001

The results demonstrated that HuM6-1B9 significantly augmented NK cell-mediated cytotoxicity in all tested breast cancer cell lines, including MCF7 (Fig. 4A), MDA-MB-453 (Fig. 4B), MDA-MB-231 (Fig. 4C), and HCC38 (Fig. 4D) compared to the medium control. No enhancement of cytotoxicity was observed in the presence of hIgG. Notably, MDA-MB-231 cells exhibited resistance to NK cell-mediated cytotoxicity in the absence of HuM6-1B9. However, treatment with HuM6-1B9 markedly increased the susceptibility of MDA-MB-231 cells to NK cell killing (Fig. 4C), indicating that HuM6-1B9 can effectively overcome NK cell resistance in this aggressive breast cancer cell line.

### HuM6-1B9 mediates NK cell degranulation and IFN-γ secretion against TNBC cell line

**Fig. 5:**
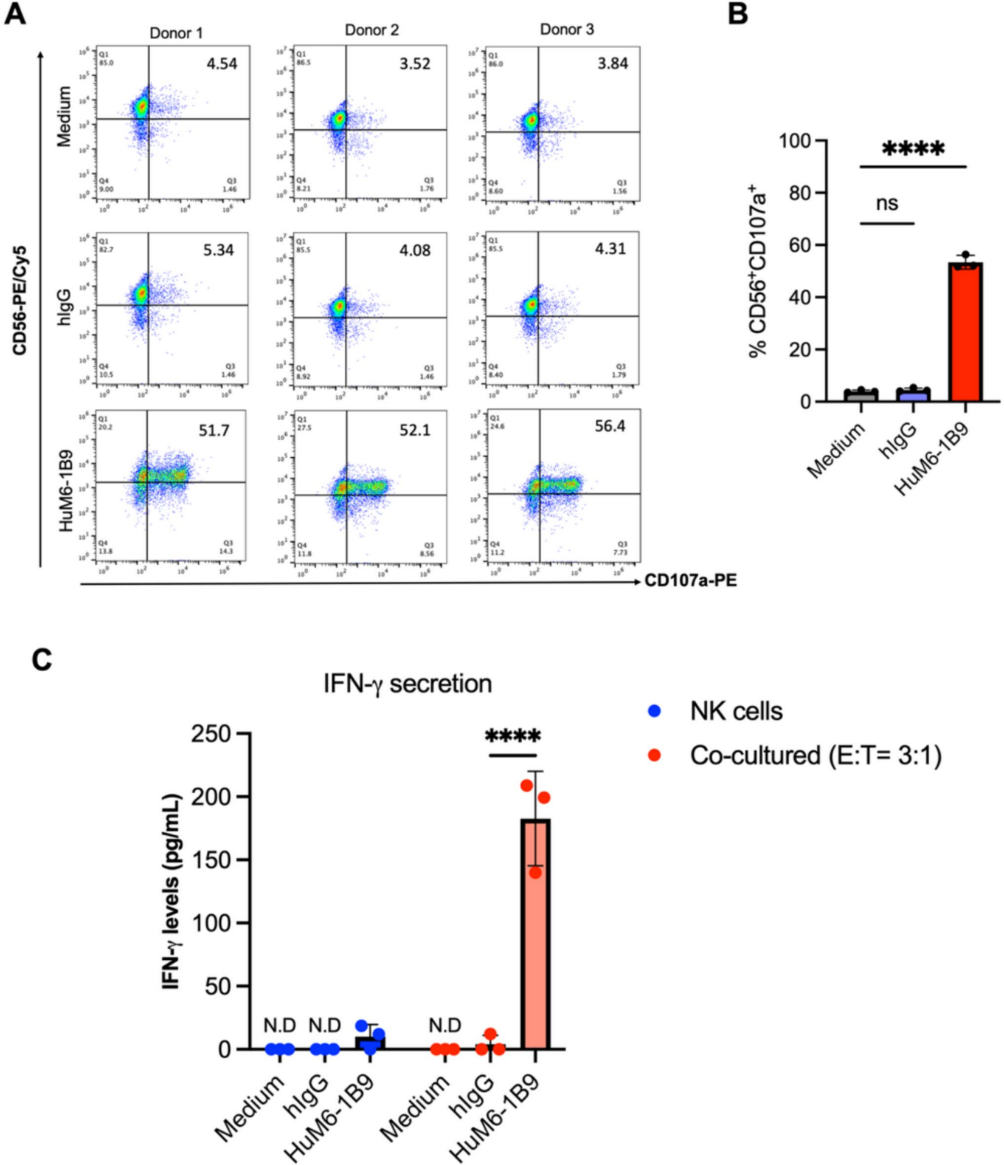
HuM6-1B9 enhances NK cell degranulation and IFN-γ secretion via ADCC. MDA-MB-231 target cells were co-cultured with primary NK cells in the presence of HuM6-1B9, hIgG, or medium alone. Degranulated NK cells (CD56^+^CD107a^+^) were assessed using flow cytometry. (**A**) Representative dot plot showing CD107a expression in CD56^+^ NK cells from individual donors. (**B**) Quantification of CD56^+^CD107a^+^ (%) was presented as bar graphs across different conditions. Data are represented as mean ± SD from three independent healthy donors. Statistical significance was determined by one-way ANOVA with Dunnett’s post hoc test: ns, *P* > 0.05; ****P ≤ 0.0001. (**C**) Levels of IFN-γ (pg/mL) secreted into the cultured supernatant from three different donors. Data are presented as mean ± SD. Statistical significance was determined by two-way ANOVA followed by Fisher’s test: ****P ≤ 0.0001. N.D indicates IFN-γ levels below the detection limit

MDA-MB-231 cell line was used as target cells to evaluate the enhancement of NK cell activity by HuM6-1B9. The MDA-MB-231 cells were co-cultured with primary NK cells in E:T ratio of 3:1 in the presence of HuM6-1B9 or hIgG or medium alone. After 4 h of co-culture, cells were harvested and stained with PE/Cy5-anti-CD56 mAb. The degranulation of NK cells was determined using PE-anti-LAMP-1 mAb. Flow cytometric analysis was performed to quantify the percentage of CD56^+^CD107a^+^ across three different healthy donors (Fig. 5A). The results demonstrated that HuM6-1B9 significantly promoted NK cell degranulation to against MDA-MB-231 cells, compared to the medium control (Fig. 5B). Additionally, co-culturing of primary NK cells with MDA-MB-231 cells in the presence of HuM6-1B9 resulted in a significant increase in IFN-γ secretion in culture supernatant compared with the hIgG control. In contrast, co-culture of NK cells with HuM6-1B9 alone yielded only low levels of IFN-γ secretion in two healthy donors (Fig. 5C).

### HuM6-1B9 exhibits NK cell-mediated apoptosis in TNBC cell line

**Fig. 6:**
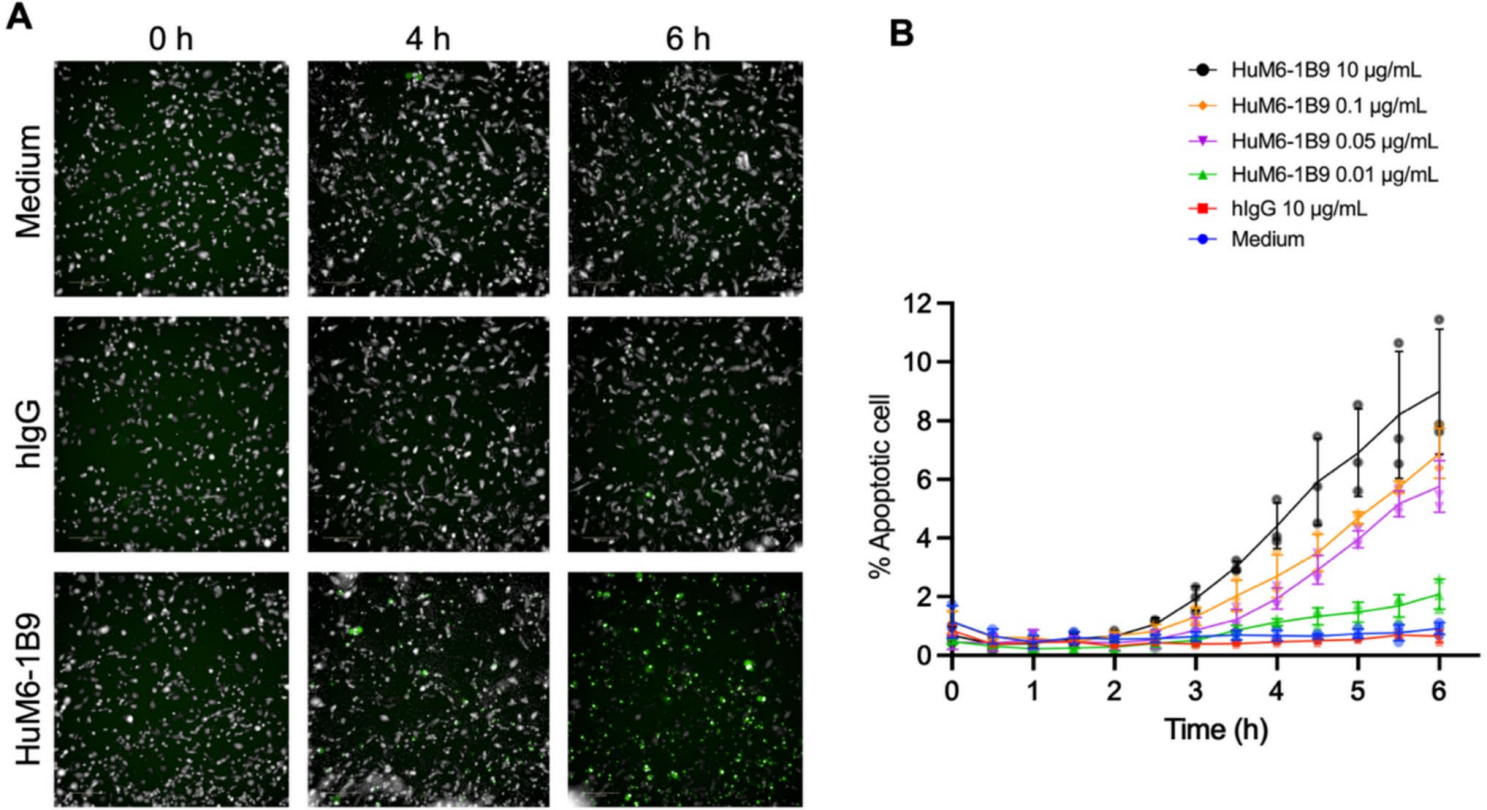
NK cell-mediated cytotoxicity activity induces apoptosis in TNBC cells. (**A**) Representative images of caspase-3/7 activity in target cells at 0, 4, and 6 h following co-culture with NK cell in the absence of antibody (Medium) or in the presence of HuM6-1B9 or hIgG at 10 μg/mL. (**B**) The graph illustrates the apoptotic cell (%) (mean ± SD) in each time points. The experiment was conducted in triplicate, and apoptotic cell (%) was calculated as the ratio of green-fluorescent cell to the total cell number. Live-cell videos of apoptotic activity are demonstrated in supplementary videos

MDA-MB-231 cells were seeded in 96-well flat-bottom plate and incubated overnight. The following day, the cells were labeled with a caspase-3/7-activity dye (green-fluorescent color) and co-cultured with primary NK cells in the presence or absence of HuM6-1B9. After a 6 h incubation, a marked increase in the number of green-fluorescent target cells were observed in the HuM6-1B9-treated group compared to the control (Fig. 6A), indicating enhanced induction of TNBC apoptosis by NK cells in the presence of the HuM6-1B9. Quantitative analysis of caspase-3/7-activity-positive cells further demonstrated that HuM6-1B9 significantly increased the percentage of apoptotic target cells in a time- and concentration-dependent manner (Fig. 6B). These findings suggest that HuM6-1B9 potentiates NK cell-mediated cytotoxicity by promoting apoptosis in resistant breast cancer cells.

### HuM6-1B9 enhances ADCC via FcγRIII interaction without modulating MIC-A/B expression or synergizing with PD-1 blockade

**Fig. 7:**
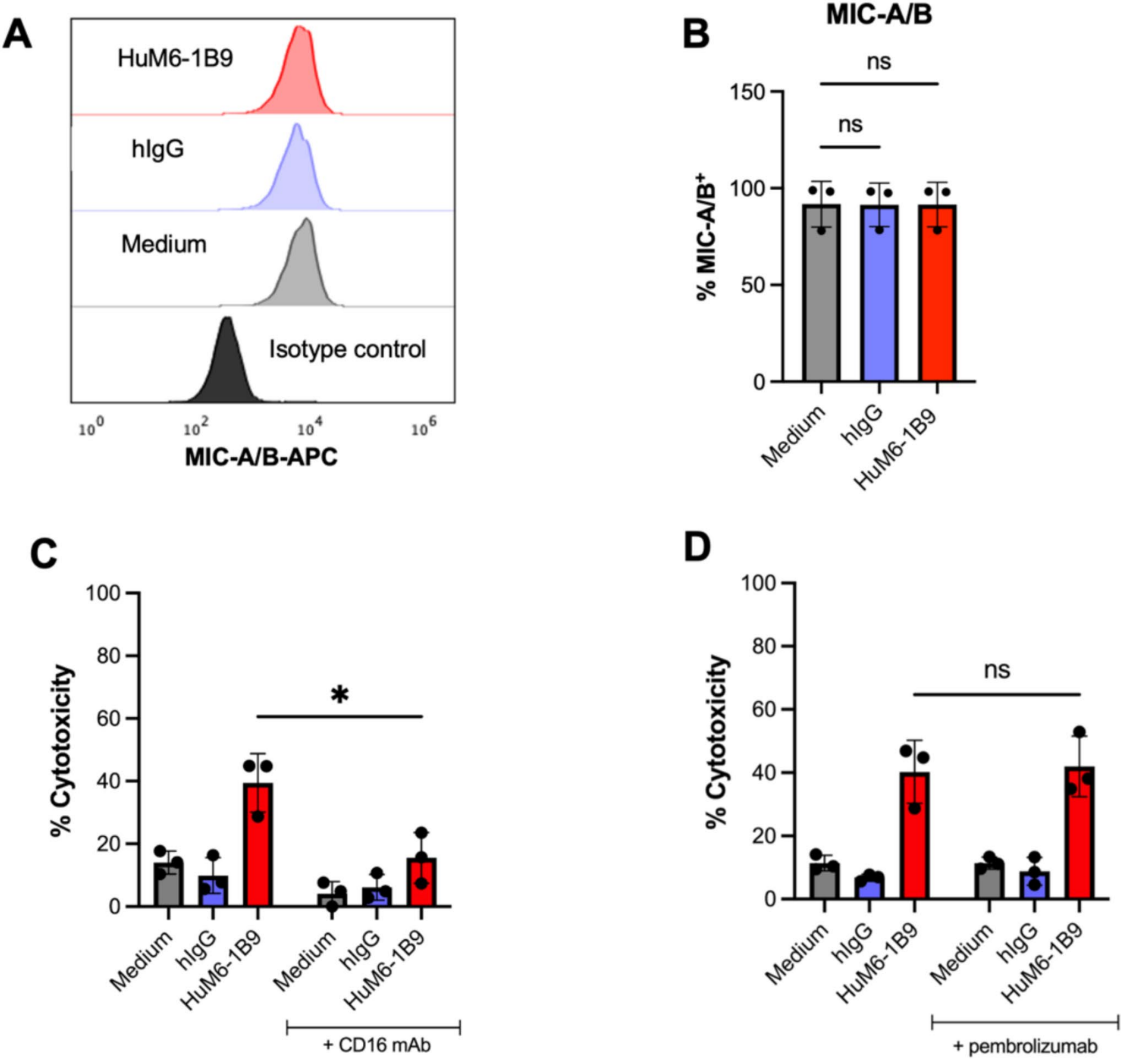
ADCC enhancement by HuM6-1B9 is FcγRIII-dependent and independent of MIC-A/B modulation or PD-1 blockade. (**A**) Histogram of MIC-A/B expression on MDA-MB-231 after CD147 blockade by HuM6-1B9. (**B**) The bar graph (mean ± SD) represents MIC-A/B^+^ cells (%) in each condition from three independent experiments. Statistical significance was determined by one-way ANOVA with Dunnett’s post hoc test: ns,* P* > 0.05. (**C**) Bar graph (mean ± SD) represents the cytotoxicity (%) of ADCC inhibition by anti-CD16 mAb. (**D**) Effect of HuM6-1B9 and anti-PD-1 combination in ADCC activity against MDA-MB-231. (**C–D**) Data were performed in three independent experiments, and statistical analysis was determined using two-way ANOVA followed by Fisher’s test: ns, *P* > 0.05; **P ≤ 0.01

MDA-MB-231 cells were treated with HuM6-1B9 for 24 h to assess whether CD147 blockade affected the expression of MIC-A/B on the cell surface. Flow cytometric analysis showed that MIC-A/B expression levels on HuM6-1B9-treated cells were comparable to those of cells cultured in medium alone or hIgG (Fig. 7A). Consistently, the percentage of MIC-A/B⁺ cells did not significantly differ among conditions (Fig. 7B). To further investigate the cytotoxicity mechanism underlying ADCC enhancement, an FcγRIII-blocking assay was performed using an anti-CD16 mAb. Blocking FcγRIII markedly reduced NK cell-mediated cytotoxicity (Fig. 7C), indicating that HuM6-1B9 enhances ADCC primarily through Fc–FcγRIII interactions. Additionally, the effect of combining HuM6-1B9 with immune checkpoint blockade in ADCC enhancement was evaluated. The result showed that pretreatment of NK cells with pembrolizumab (anti-PD-1) prior to co-culture with HuM6-1B9–treated MDA-MB-231 cells did not further increase ADCC activity (Fig. 7D).

### HuM6-1B9 does not inhibit the migration and invasion of the MDA-MB-231 breast cancer cell line

**Fig. 8:**
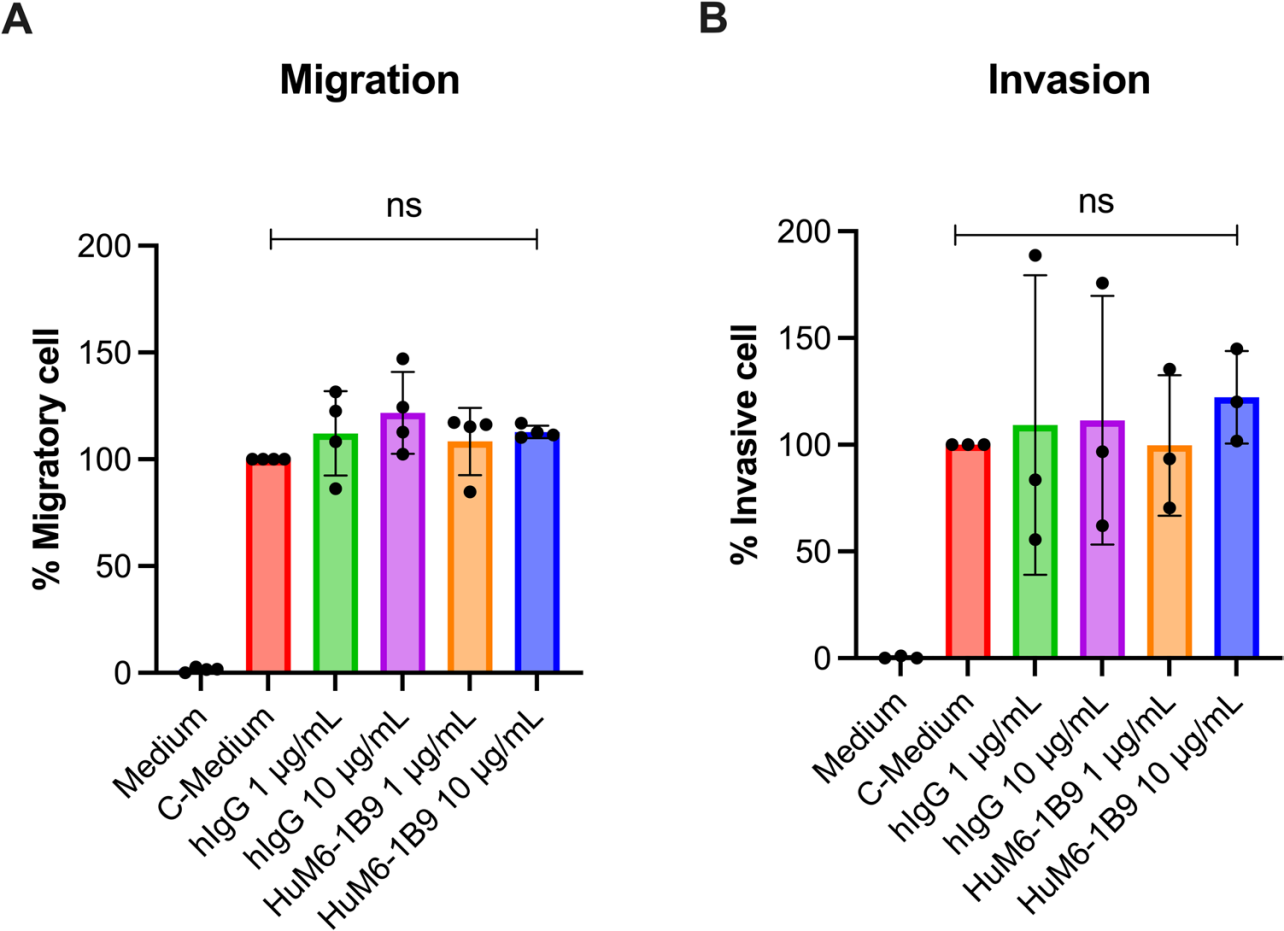
Effect of HuM6-1B9 on MDA-MB-231 migration and invasion. A transwell assay was performed to evaluate the inhibitory effect of HuM6-1B9 on (**A**) cell migration and (**B**) cell invasion of MDA-MB-231. The migratory or invasive cell (%) is presented relative to the C-Medium control. The migration assay was performed in quadruplicate, and the invasion assay was performed in triplicate. Bar graphs represent the mean ± SD. Statistical analysis was performed using one-way ANOVA followed by Tukey’s multiple comparison test: ns, *P* > 0.05

Transwell migration and invasion assays were conducted to examine the inhibitory effect of HuM6-1B9 on the motility of the TNBC cell line (MDA-MB-231). The results demonstrated that the percentage of migratory MDA-MB-231 cells in the presence of HuM6-1B9 at concentrations of 1 and 10 μg/mL was not significantly different compared to the C-Medium or hIgG control (Fig. 8A). Furthermore, no inhibitory effect on cell invasion was observed in the presence of HuM6-1B9 (Fig. 8B). These findings suggest that targeting CD147 with HuM6-1B9 does not affect the migration and invasion of MDA-MB-231 breast cancer cells.

## Discussion

Previous studies have highlighted the roles of CD147 [12], CD146 [6], and CD73 [9] in tumor development, progression, and therapeutic resistance, underscoring their potential as therapeutic targets. In the present study, multiple breast cancer subtypes were investigated, including MCF7 (hormone receptor-positive), MDA-MB-453 (HER2-positive), MDA-MB-231, and HCC38 (both TNBC). Within the TNBC group, MDA-MB-231 was selected as a representative of the mesenchymal subtype originating from metastatic lesions, whereas HCC38 was chosen to exemplify the basal-like 1 subtype derived from infiltrating carcinoma. Our results revealed distinct expression profiles of these markers across various breast cancer cell lines, emphasizing the molecular heterogeneity among subtypes. CD146 expression was detected exclusively in MDA-MB-231, and HCC38 cells, TNBC lines, but was absent in MCF7 and MDA-MB-453 cells. This suggests a possible association between CD146 expression and the aggressive phenotype of TNBC [7, 10]. CD73 was minimally expressed in both MCF7 and HCC38 cells, with markedly higher expression observed in MDA-MB-231 cells, reinforcing its potential as a target in mesenchymal TNBC subtype. Notably, CD147 was uniformly expressed across all cell lines, supporting its utility as a broadly applicable target for breast cancer treatment.

Measuring the K_D_ of a therapeutic antibody by titration-based flow cytometry provides quantitative insight into its binding to native antigens on breast cancer cells. This method preserves physiological antigen expression and conformation, aiding in the selection of antibodies with optimal binding for effective immune activation. Given the heterogeneity of breast cancer, such analysis is crucial for identifying candidates with broad applicability. K_D_ of HuM6-1B9 was determined by fitting binding curves using nonlinear regression [25]. The binding affinity analysis revealed that HuM6-1B9 demonstrated strong binding to all tested breast cancer cell lines, with *K*_*D*_ ranging from 4.523 to 7.910 nM. The highest affinity was observed in HCC38 cells (*K*_*D*_ = 4.523 nM), consistent with their high surface expression of CD147. Despite slightly lower affinities for MCF7 and MDA-MB-453 cells, the nanomolar K_D_ values reflect strong binding interactions. Notably, these values are comparable to those of clinically approved antibodies such as trastuzumab and pertuzumab [26], positioning HuM6-1B9 as a viable candidate for therapeutic antibody.

Immunotherapy has emerged as a promising strategy in oncology, demonstrating significant improvements in overall and progression-free survival for various cancers [27]. Among immunotherapeutic mechanisms, ADCC plays a critical role in mediating the elimination of tumor cells. Previous study has demonstrated that HuM6-1B9 effectively mediates ADCC against MDA-MB-231 cells cultured in a two-dimensional (2D) system, using PBMCs as effector cells [24]. However, conventional 2D culture systems lack the structural complexity and extracellular matrix interactions characteristic of native tumor architecture and its associated microenvironment [28]. Accordingly, the present study evaluated the ADCC activity of HuM6-1B9 using a 3D Matrigel-based spheroid model to better recapitulate in vivo tumor conditions. Co-culturing PBMCs with TNBC spheroids in the presence of HuM6-1B9 significantly reduced spheroid cell viability, indicating that HuM6-1B9 retains its ADCC-inducing capability in a more physiologically relevant 3D context. Additionally, we examined PBMC infiltration into TNBC spheroids. As shown in Fig. S1, PBMCs successfully infiltrated the spheroids, potentially guided by chemoattractants secreted by the tumor cells [29, 30]. Notably, after 6 h of co-culture, HuM6-1B9 treatment appeared to impair PBMC infiltration, possibly due to reduced spheroid viability and diminished chemoattractant production. While PBMCs were capable of mediating TNBC cell death, it remains unclear whether NK cells, a major cytotoxic subset within PBMCs, were specifically responsible for this effect. To address this, we performed flow cytometry-based ADCC assays using isolated NK cells as effectors to evaluate the functional efficacy of HuM6-1B9. The results confirmed HuM6-1B9-mediated enhancement of NK cell cytotoxicity across all breast cancer cell lines tested. Interestingly, MDA-MB-231 cells exhibited the lowest baseline NK-mediated lysis (approximately 10%), which may be attributed to higher expression of MHC class I molecules, key ligands that inhibit NK cell activity. In contrast, MCF7 cells showed elevated NK-mediated cytotoxicity even in the absence of antibody, possibly due to higher expression of activating NK ligands [31]. Notably, HCC38 cells exhibited higher NK cell-mediated cytotoxicity in the medium control compared with MDA-MB-231, despite the latter expressing higher levels of MHC class I. This observation may reflect intrinsic differences between these TNBC cell lines, such as variations in death receptor expression, which could influence apoptotic susceptibility [32].

Mechanistically, NK cell-mediated cytotoxicity primarily involves the induction of apoptosis upon recognition of antibody-coated target cells [33]. In our study, HuM6-1B9 significantly enhanced ADCC in MDA-MB-453 cells relative to MCF7, MDA-MB-231, and HCC38 cells. This may be due to lower levels of antiapoptotic proteins in MDA-MB-453 cells [34], rendering them intrinsically more susceptible to ADCC-mediated killing. Furthermore, to validate the observed enhancement of ADCC in MDA-MB-231 cells by HuM6-1B9 and to directly visualize the cytotoxic effect detected in the flow cytometric-based ADCC assay, we subsequently performed an apoptosis assay using caspase-3/7-activity staining and live-cell imaging. The live-cell imaging assay corroborated the flow cytometric data, demonstrating that HuM6-1B9 effectively augmented NK cell-mediated killing of MDA-MB-231 cells. Moreover, co-culturing of NK cells and HuM6-1B9-treated MDA-MB-231 cells significantly enhanced the degranulation of NK cells and the releasing of IFN-γ in culture supernatant, indicating that HuM6-1B9 plays a critical role in ADCC enhancement. In addition, our previous study demonstrated that treatment of MDA-MB-231 with HuM6-1B9 did not directly induce target cell death [24]. Additionally, the alteration effect of HuM6-1B9 toward NK cell-activating ligand (such as MIC-A/B) was observed. Our data demonstrated that the expression of MIC-A/B on TNBC cell surface was not alter by HuM6-1B9. Preservation of MIC-A/B expression ensures that HuM6-1B9 does not interfere with endogenous NK cell-mediated tumor recognition and killing but rather enhances cytotoxicity. These convergent results provide strong evidence for the efficacy of HuM6-1B9 in enhancing ADCC, highlighting the potential of this antibody in therapeutic strategies targeting CD147.

ADCC is mediated by the interaction of the antibody Fc region with FcγRIIIa (CD16a), an activating receptor predominantly expressed on NK cells [35]. CD16a is a potent inducer of NK cell activation and degranulation [36, 37]. In the present study, blockade of FcγRIII on NK cells with CD16 mAb prior to co-culture with HuM6-1B9-treated target cells reduced cytotoxicity. Consistently, CD16 blockade significantly diminished HuM6-1B9-induced ADCC in our previous study [24], confirming that HuM6-1B9 functions primarily via CD16-mediated pathways. Beyond direct cancer cell lysis, NK cell activation also contributes to the recruitment of dendritic cells into the tumor microenvironment, thereby playing a multifaceted role in cancer immune control [38]. Importantly, HuM6-1B9 exhibited no cytotoxicity against normal PBMCs [24], indicating a targeted mechanism that spares healthy cells and underscores its potential safety in clinical applications.

The interplay between ADCC and immune checkpoint pathways offers important insight into the observed results. Immune checkpoint molecules such as PD-1 are upregulated on NK cells, particularly under inflammatory conditions driven by IL-18 in TNBC [39]. Furthermore, TNBC cell lines commonly express higher levels of PD-L1 compared to other subtypes [40–42], contributing to impaired NK cell cytotoxicity. In advanced TNBC, response rates to PD-1/PD-L1 inhibitors are typically low, especially in tumors with minimal PD-L1 expression [43]. A synergistic effect between HuM6-1B9 and anti-PD-1 was not observed in the present study. This outcome may be attribute to the low basal expression of PD-1 on resting NK cells [44]. Although the combination of HuM6-1B9 and immune checkpoint blockade did not directly enhance ADCC under the current experimental condition, such a therapeutic strategy may still potentiate NK cell activity through ADCC in the exhausted NK cell-status of TNBC patients, thereby offering a strategy to overcome immune suppression in TNBC's immunologically "cold" tumor microenvironment.

CD147 has been implicated in cancer cell migration and invasion through the induction of matrix metalloproteinases (MMPs), including MMP-2 and MMP-9 [45, 46]. Surprisingly, HuM6-1B9 treatment did not affect migration or invasion in MDA-MB-231 cells. This may be explained by the antibody’s epitope specificity; HuM6-1B9 binds to the ^31^EDLGS^35^ epitope on CD147 [47], which lies outside the MMP-inducing domains [48–50]. Furthermore, HuM6-1B9 binds to a distinct epitope of CD147 that does not participate in the interactions with adhesion molecules such as integrin β1 subunit [51]. The binding specificity of HuM6-1B9 likely prevents the activation of pro-metastatic signaling pathways, such as those mediated by integrin or EGFR interactions, which are known to drive MMP expression and EMT [48, 49, 51]. By avoiding these signaling cascades, HuM6-1B9 offers a significant therapeutic advantage, as promoting cell migration in TNBC could undermine the cytotoxic benefits of antibody-based therapies. Indeed, many mAbs have been associated with unintended pro-invasive effects. For example, EGFR blockade may lead to compensatory upregulation of alternative pathways that facilitate tumor invasiveness and EMT. In colorectal cancer, treatment with cetuximab has been linked to increased *MET* amplification, a change associated with enhanced metastatic potential [52]. In contrast, HuM6-1B9 exerts its antitumor activity through an ADCC-driven mechanism without promoting cell migration, thereby supporting a targeted strategy for tumor elimination without exacerbating metastatic risk. This is particularly relevant for TNBC, where metastasis remains the leading cause of cancer-related mortality.

Despite demonstrating that HuM6-1B9 enhances NK cell-mediated ADCC in TNBC cell lines and is efficacious in 3D spheroid models, this study has several limitations. First, the lack of CD147-deficient TNBC cell lines precluded direct confirmation of target specificity in breast cancer-derived models. Instead, reliance on prior THP-1 knockout data provided mechanistic plausibility [21] but did not directly validate the findings in TNBC. Second, the study did not include a direct comparison with clinically approved ADCC-enhancing antibodies (e.g., trastuzumab), which would have been valuable for benchmarking translational relevance. Finally, although 3D spheroid models offer a more physiologically relevant environment than traditional 2D cultures, in vivo studies remain essential to fully establish safety, efficacy, and potential synergy within the tumor microenvironment. These limitations underscore important next steps in the preclinical development of HuM6-1B9.

In conclusion, HuM6-1B9 targeting CD147 exhibits strong therapeutic potential in TNBC by enhancing NK cell-mediated ADCC and apoptosis, even in the context of high MHC class I expression. Beyond validating ADCC activity in 3D spheroid models, this study provides mechanistic evidence that underscores the translational relevance of HuM6-1B9. These results support its further development as a candidate for combination immunotherapies, particularly with immune checkpoint inhibitors. Alongside CD147, Trop-2 has emerged as a clinically validated target in TNBC, exemplified by the FDA-approved antibody–drug conjugate Sacituzumab govitecan (Trodelvy), which has demonstrated survival benefits in patients with advanced disease. Nevertheless, not all TNBC patients benefit from Trop-2-directed therapy, and resistance or heterogeneous Trop-2 expression may limit durable responses [53]. Thus, CD147-directed therapy may complement Trop-2 ADCs to broaden therapeutic coverage, overcome resistance, and provide additional benefit in patients with aggressive TNBC. Future in vivo studies using xenograft and patient-derived models are warranted to confirm efficacy, safety, and therapeutic synergy within the tumor microenvironment.

## Supplementary Information

Below is the link to the electronic supplementary material.

Supplementary file1 (MP4 1009 kb)

Supplementary file2 (MP4 934 kb)

Supplementary file3 (MP4 575 kb)

Supplementary file4 (DOCX 6740 kb)

## Data Availability

Data are available upon reasonable request.
