# Supplementary material for: Overcoming NK cell resistance in triple-negative breast cancer via adcc with a humanized anti-CD147 antibody
Source: Cancer Immunol Immunother. 2025 Oct 22;74(11):342. doi: 10.1007/s00262-025-04203-z (PMC12545948; doi:10.1007/s00262-025-04203-z)
Supplement: Supplementary file 4 — Supplementary file4 (DOCX 6740 kb) [file 262_2025_4203_MOESM4_ESM.docx]

**Supplementary materials**

**Fig. S1.** Assessment of PBMC infiltration into TNBC spheroids. MDA-MB-231 cells were pre-stained with Hoechst 33342 prior to spheroid formation. PBMCs were labeled with CMFDA dye. TNBC spheroids (blue) were co-cultured with CMFDA-labeled PBMCs (green) in the presence or absence of HuM6-1B9 (10 μg/mL) or cultured without PBMCs as a control. PBMC infiltration into TNBC spheroids was analyzed using the Operetta CLS™ high-content analysis system. (A) Line graph showing the relative PBMC infiltration into spheroids over time (n=3). (B) Representative Z-stack images comparing PBMC infiltration into TNBC spheroids in the absence or presence of HuM6-1B9.

**
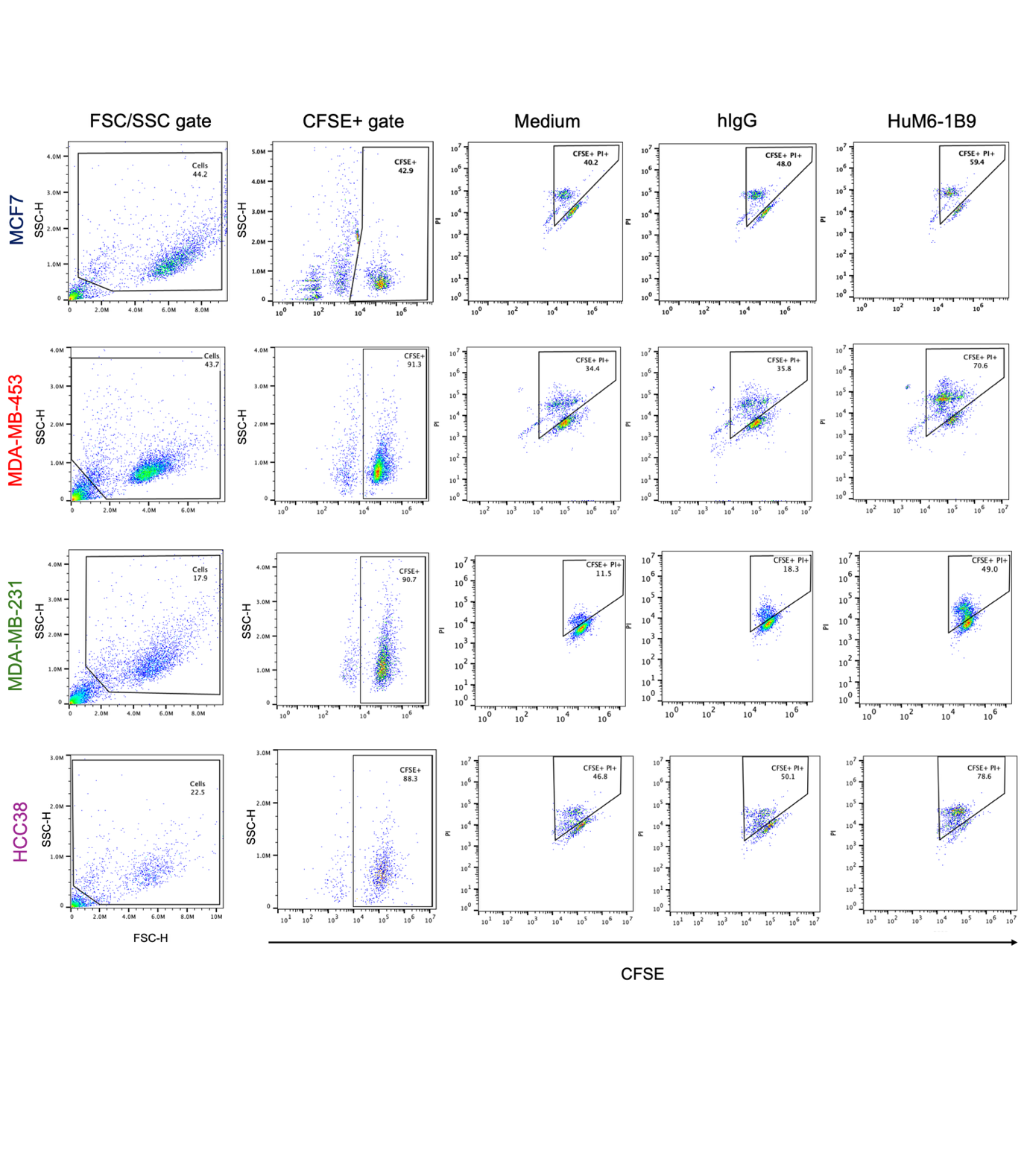
**

**Fig. S2.** CFSE-labeled target cells were co-cultured with primary NK cells (effector cells) in the presence of HuM6-1B9 or hIgG (10 μg/mL), or in the absence of antibody (Medium). Target cell death was assessed by PI staining. CFSE-positive target cells (CFSE⁺ gate) were gated based on the FSC/SSC plot.
